# Investigation on C_f_/PyC Interfacial Properties of C/C Composites by the Molecular Dynamics Simulation Method

**DOI:** 10.3390/ma12040679

**Published:** 2019-02-25

**Authors:** Yuan Zhou, Tianyuan Ye, Long Ma, Zixing Lu, Zhenyu Yang, Shouwen Liu

**Affiliations:** 1 Beijing Institute of Spacecraft Environment Engineering, Beijing 100091, China; sacalier@163.com (Y.Z.); 15510009171@163.com (L.M.); sleigh00@163.com (S.L.); 2 Beijing Key Laboratory of Environment & Reliability Test Technology for Aerospace Mechanical & Electrical Products, Beijing 100091, China; 3 School of Aeronautical Science and Engineering, Beihang University, Beijing 100091, China; luzixing@buaa.edu.cn (Z.L.); zyyang@buaa.edu.cn (Z.Y.)

**Keywords:** interphase, C/C composite, molecular dynamics simulation

## Abstract

In this paper, a molecular dynamics (MD) simulation model of carbon-fiber/pyrolytic-carbon (C_f_/PyC) interphase in carbon/carbon (C/C) composites manufactured by the chemical vapor phase infiltration (CVI) process was established based on microscopic observation results. By using the MD simulation method, the mechanical properties of the C_f_/PyC interphase under tangential shear and a normal tensile load were studied, respectively. Meanwhile, the deformation and failure mechanisms of the interphase were investigated with different sizes of the average length ${\bar{L}}_{a}$ of fiber surface sheets. The empirical formula of the interfacial modulus and strength with the change of ${\bar{L}}_{a}$ was obtained as well. The shear properties of the isotropic pyrolysis carbon (IPyC) matrix were also presented by MD simulation. Finally, the mechanical properties obtained by the MD simulation were substituted into the cohesive force model, and a fiber ejection test of the C/C composite was simulated by the finite element analysis (FEA) method. The simulation results were in good agreement with the experimental ones. The MD simulation results show that the shear performance of the C_f_/PyC interphase is relatively higher when ${\bar{L}}_{a}$ is small due to the effects of non-in-plane shear, the barrier between crystals, and long sheet folding. On the other hand, the size of ${\bar{L}}_{a}$ has no obvious influence on the interfacial normal tensile mechanical properties.

## 1. Introduction

Carbon/carbon (C/C) composites are one of the most important ceramic matrix composites, which are often used to manufacture thermal protection structures in the aerospace field [1]. C/C composites are usually prepared by depositing a pyrolytic carbon (PyC) matrix on carbon fiber preforms using chemical vapor phase infiltration (CVI) or chemical vapor phase deposition (CVD) processes [2,3]. In the early stage of PyC deposition, due to the induction effect of the fiber surface structure, the microstructure near the fiber surface will form a transition interphase, which is different from either the carbon fiber or the isotropic pyrolysis carbon (IPyC) matrix, and should be treated as a separate third phase. The performance of the interphase has a close relationship with the toughening mechanism of fiber-reinforced ceramic matrix composites (such as crack propagation delay or deflection, fiber bridging toughening, interface debonding, and fiber pull out), and has a great influence on the composite mechanical properties and the stress–strain behaviors [4,5,6]. Therefore, the interfacial properties of ceramic matrix composites have been one of the focuses of scholars’ study.

Since the thickness of the interphase between two phase materials is usually at the nanometer level, the molecular dynamics (MD) simulation method is often adopted to study the deformation mechanism, mechanical properties, and failure behavior of the interphase under a certain external load. Pishehvarz et al. [7] used the MD simulation method to study the interfacial properties between modified graphene-based nanosheets and conductive polymers, focusing on the interaction between the interphase and the reinforcement brought about by it, and obtained good results that were consistent with experimental data. Alian et al. [8] evaluated the influence of waviness and agglomeration of curved and agglomerated carbon nanotubes (CNTs) on the interfacial strength of thermoset nanocomposites and upon their load transfer capability by using the MD simulation method, focusing on the influence of the curvature and diameter of carbon nanotubes on the interfacial shear strength. Elkhateeb et al. [9] used the MD method to simulate the crack propagation along the interface of Ti6Al4V/TiC in titanium metal matrix composites under Mode-I and II loadings and at different temperatures, and obtained the traction-separation relationship, which was used to parameterize the cohesive zone model (CZM) for modeling the interphase in finite element analysis. A good agreement was achieved between the stress–strain results obtained from simulation and the experimental data under the same conditions. Nouranian and Jang et al. [10,11] used MD simulation to perform an investigation on the role of liquid vinyl ester (VE) resin monomer interactions with the surface of both pristine and oxidized vapor-grown carbon nanofibers (VGCNFs). VGCNF–matrix adhesion together with interphase stiffness were analyzed in both pristine and oxidized VGCNF surface situations, and insight into carbon nanofiber–matrix interactions were successfully provided to facilitate multiscale material design. However, the existing MD simulation work on graphite-like structures mainly focuses on the properties of carbon nanotubes and graphene materials [12,13,14,15], and there are few reports on the interfacial mechanical behavior in fiber-reinforced ceramic composites.

Based on the microscopic observation results of the carbon-fiber/pyrolytic-carbon (C_f_/PyC) interphase in C/C composites manufactured by the CVI process, an MD simulation model of the C_f_/PyC interphase was established in this paper. On this basis, the mechanical properties and failure mechanism of the C_f_/PyC interphase under tangential shear and a normal tensile load, as well as the strengthening mechanism with different sizes of the average length ${\bar{L}}_{a}$ of fiber surface sheets, were studied. Finally, the interfacial and IPyC matrix properties obtained by the MD simulation were used in the finite element analysis (FEA) of a fiber ejection test on a C/C composite, and the effectiveness of the MD simulation results was verified.

## 2. Materials and Methods

### 2.1. Microstructure of the C_f_/PyC Interphase Manufactured by the CVI Process

In order to accurately obtain the mechanical properties of the C_f_/PyC interphase, the MD model used in the simulation must truly reflect the C_f_/PyC interphase’s topography. Based on the microscopic observation results in the literature [5,6,16], the microstructural characteristics of the C_f_/PyC interphase manufactured by the CVI process are summarized as follows. First, the polyacrylonitrile (PAN) carbon fiber is consistent with the classic skin-core structure [17], and the graphite sheets in the fiber surface have the highest anisotropy and flatness values. Second, the carbon fiber and the pyrolytic carbon nearby are composed of graphite sheets, with no obvious interface. Third, in the pyrolysis carbon matrix far away from the fiber surface, the interphase presents an IPyC structure: there is no obvious lamellar structure or preferred orientation, and the carbon structure shows isotropic characteristics. Fourth, between the fiber surface and the isotropic pyrolytic carbon, there is a thin transition area induced by the fiber surface’s structure. In this area, the distribution of each sheet is discrete, but generally they are approximately along the direction of the fiber surface, showing high anisotropy and flatness. The change in the PyC structure from the fiber surface to the IPyC is asymptotic. Fifth and finally, for high-modulus carbon fiber, which has a larger average size of graphite sheets in the fiber surface (such as T50 carbon fiber), the graphitization degree of the fiber surface is higher, as well as the orientation of graphite sheets in the transition region. Meanwhile, the transition region is thicker (about 15 nm). On the other hand, for high-strength carbon fiber, which has a smaller average size of graphite sheets in the fiber surface (such as T300 carbon fiber), the graphitization degree in the transition region is lower, with smaller graphite sheets. For example, in T300 carbon fiber, the longitudinal length of the graphite sheets in the fiber surface is about 2~4 nm, while the longitudinal length in the transition regions nearby is about 3~5 nm, and the transition region thickness is about 8 nm [18].

### 2.2. Establishment of the MD Simulation Model

#### 2.2.1. MD Simulation Model for the C_f_/PyC Interphase

The LAMMPS MD simulator was used to construct the interphase model and perform the simulation. Since the main purpose of this paper was focused on the interfacial properties during normal tensile and longitudinal shear, the MD simulation model only considered the configuration along the longitudinal and radial directions of the fiber (the x and z directions in the model, respectively). In order to investigate the influence of the fiber surface’s average sheet length on the morphology of the C_f_/PyC interphase region, the PyC deposition in the CVI process was simulated by MD simulation under the following assumptions.

**Figure 1:**
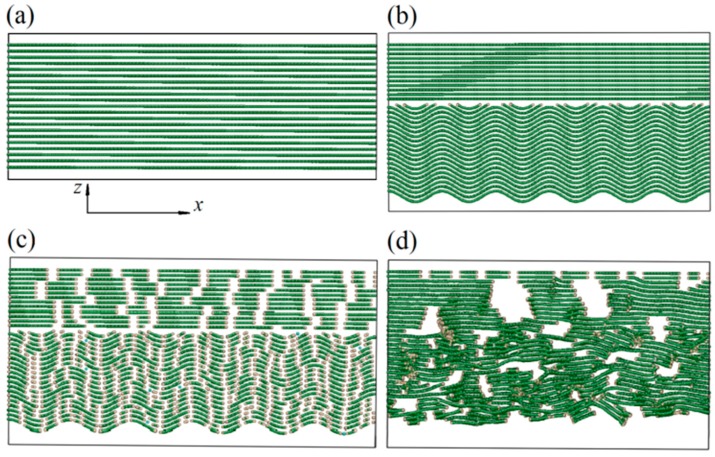
Establishment of the molecular dynamics (MD) model of the C_f_/PyC interphase. (**a**) initial perfect hexagonal graphite structure; (**b**) deformed using the cosine function; (**c**) after cutting; (**d**) final structure.

**Figure 2:**
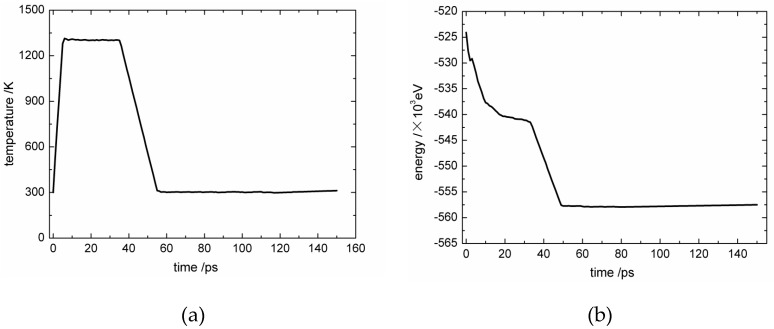
The change in system temperature and total energy with time during annealing: (**a**) system temperature; (**b**) system total energy.

First, an MD model of perfect hexagonal graphite with 21 layers was established, with the distance between the graphene sheets equal to 3.4 angstrom (as shown in Figure 1a). Each laminate had 100 six-carbon rings in the x direction and 8 in the y direction. The AIREBO potential function was used to describe the covalent bonding and van der Waals force [19], and the cut-off distance for long-range interactions was set to 13.6 angstrom. Second, in order to characterize the fluctuation in the PyC direction near a carbon fiber, while satisfying the periodic requirement, it was assumed that the initial direction of graphite sheets in the PyC met the cosine function. Another 13 flat layers were added on the top as the sheets in the surface of the fiber, as shown in Figure 1b. Third, in order to reflect the random length of graphite wafers both in the carbon fiber and the PyC, the graphite sheets were cut at random positions with random lengths (1~3 atoms in the x direction and 1~4 layers in the z direction), as shown in Figure 1c. Fourth, the initial model generated in the third step was annealed once to simulate the CVI manufacturing process. Specifically, by using an NVT ensemble, the model temperature was rapidly raised from room temperature (300 K) to the typical temperature of PyC deposition in the CVI process (1300 K) with a time step of 1 fs. The temperature increment was set to 250 K, and the dynamics simulation was run for 1 ps at each intermediate temperature. Then, a relaxation of 30 ps was conducted at 1300 K. During the relaxation process, the top two layers of graphite sheets were fixed as the boundary conditions of the upper surface to reflect the constraint effect of the fiber’s internal structure. In the CVI process, the external surface on the PyC side is directly exposed to the reaction gas without any constraint. So, the bottom surface of the model adopted free boundary conditions. The other four boundary surfaces of the model used periodic boundary conditions. In the process of high-temperature relaxation, each graphite microcrystal interacted and combined with others nearby, and the position, length, and trend of the sheets changed obviously to form the growth process. The dynamics simulation at 1300 K was ended when the graphite microcrystal interacted adequately on the basis of structural features, while a long anneal time at this high temperature usually led to an obvious agglomeration effect and unreal structural features. After this, the temperature was slowly reduced to 300 K, with a temperature increment of 50 K. The dynamics simulation was also run for 1 ps at each intermediate temperature. Finally, a second relaxation of 100 ps was conducted at room temperature to form the final C_f_/PyC interphase model, as shown in Figure 1d. The changes in system temperature and total energy over time during the entire annealing process are shown in Figure 2a,b, respectively. At the end of the relaxation, the temperature and total energy of the system tended to be stable at a relatively low level, which indicated that a relatively stable MD simulation configuration of the C_f_/PyC interphase was obtained.

As can be seen from Figure 1d, the interface between the carbon fiber and the PyC is no longer obvious after relaxation. In the surface area of the carbon fiber, the graphite sheets show a high degree of anisotropy and orientation along the longitudinal direction (x direction), with a certain range of randomness in the wafer length. In the transitional region near the fiber surface, the orientation of the graphite sheets also shows a relatively high anisotropy in total; however, in each local part, the length, uniformity, and orientation of the sheets show obvious randomness. Along the z axis in the PyC region, the 2–3 layers of graphite sheets nearest to the fiber surface show the highest orientation degree. As the distance from the fiber surface increases, the randomness of the sheet length, uniformity, and orientation increases gradually. This shows a strong induction effect of the fiber surface’s structure on the orientation of nearby PyC sheets. These characteristics are consistent with the microscopic observation results described in the previous section, which indicates that the model established in this paper can accurately reflect the real structure of the C_f_/PyC interphase. After the model was generated, the average length ${\bar{L}}_{a}$ of graphite sheets in the carbon fiber surface region was recalculated statistically:

(1) $${\bar{L}}_{a}=\frac{\sum_{i=1}^{n}L_{ai}}{n}$$

where *n* is the total number of graphite sheets in the fiber surface region, and *L_ai_* is the length of sheet *i*.

**Figure 3:**
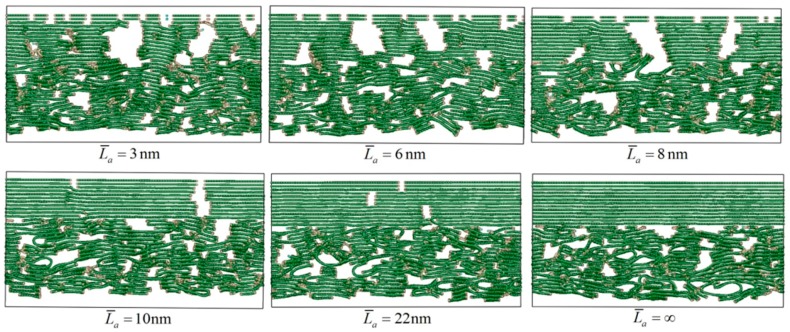
Typical C_f_/PyC interphase models with different sizes of ${\bar{L}}_{a}$.

In order to investigate the effect of different ${\bar{L}}_{a}$ on the C_f_/PyC interfacial mechanical properties, multiple MD models were established with different ${\bar{L}}_{a}$, as shown in Figure 3. In particular, due to the randomness of the established model, the calculated results of mechanical properties also have a certain dispersion, especially with small ${\bar{L}}_{a}$. In order to eliminate the influence of this randomness on the final calculation results, multiple C_f_/PyC interphase MD simulation models with the same ${\bar{L}}_{a}$ were generated when needed.

#### 2.2.2. MD Simulation Model for the IPyC Matrix

**Figure 4:**
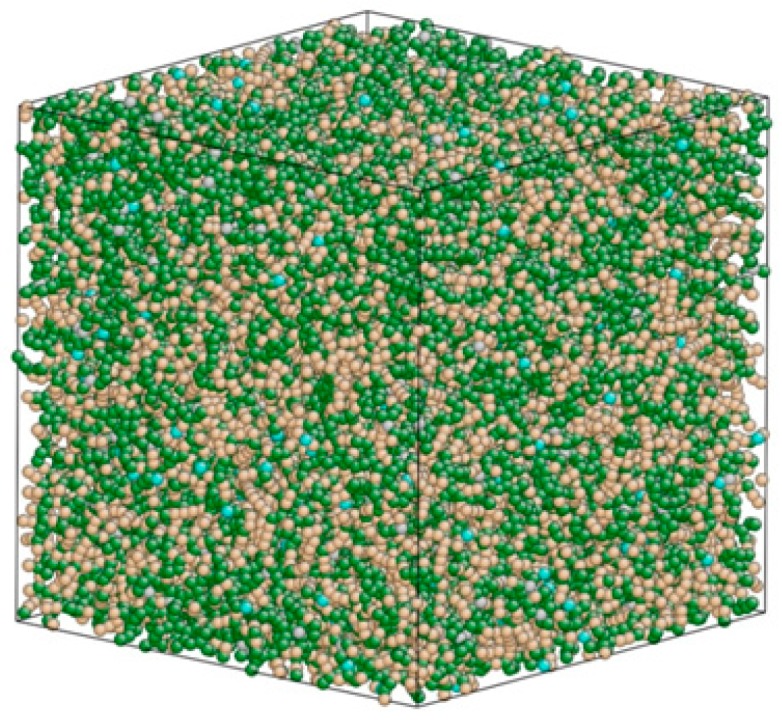
The MD simulation model of IPyC matrix with a density of 1.99 g/cm^3^.

In order to obtain the IPyC matrix shear properties to be used in the finite element analysis (FEA) of the fiber ejection test, an MD simulation model for the IPyC matrix was constructed by the following process: An MD model of perfect hexagonal graphite with 37,430 C atoms was built in a simulation box of 7.21 nm × 7.21 nm × 7.21 nm, so that the density of the model equaled 1.99 g/cm^3^. The AIREBO potential function [19] was still used with a cut-off distance of 13.6 angstrom. Using the NVT ensemble and periodic boundary conditions, the model temperature was rapidly raised from 300 K to 4500 K (above the melting point), with a time step of 1 fs. The temperature increment was set to 500 K (except the last one, which was set to 200 K), and it was run for 1 ps at each intermediate temperature. A relaxation of 200 ps was conducted at 4500 K to destroy the carbon ring structure of graphite and to obtain the discrete C atoms. After this, the temperature was slowly reduced to 300 K, with a temperature increment of 100 K (1 ps at each intermediate temperature). A second relaxation of 200 ps was conducted at 300 K to form the final IPyC matrix model, as shown in Figure 4. Due to the periodic boundary conditions and the control of the box size, the density of the model had no change during the simulation.

As shown in Figure 4, the arrangement of carbon atoms in IPyC model is disordered, containing a large number of non-hexagonal irregular carbon line structures, a small number of free C atoms, and a few complete hexagonal carbon rings. The hybrid forms of both sp^2^ and sp^3^ exist. This is consistent with the structural characteristics of the IPyC model built by Thomas et al. [20].

## 3. Results

### 3.1. MD Simulation Results of the C_f_/PyC Interphase under Normal Tensile Conditions

#### 3.1.1. Interfacial Normal Tensile Property with Different ${\bar{L}}_{a}$

**Figure 5:**
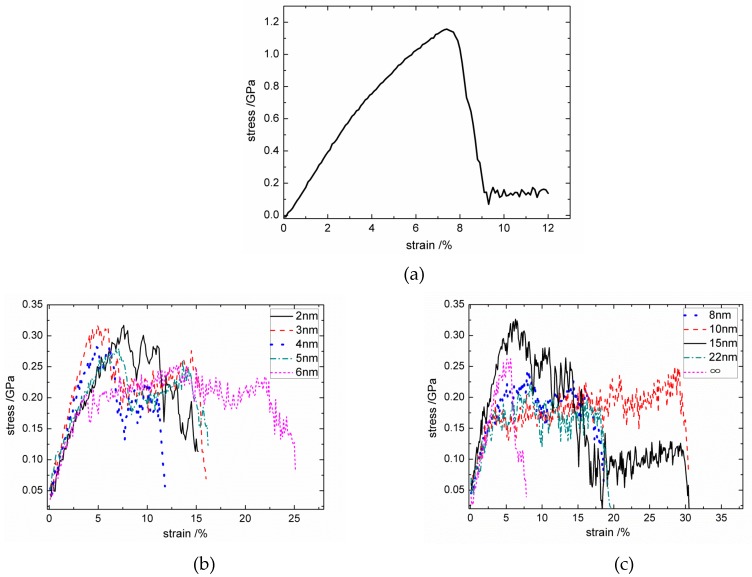
Typical normal tensile stress–strain curves: (**a**) perfect hexagonal graphite; (**b**) the C_f_/PyC interphase with ${\bar{L}}_{a}$ equal to 2–6 nm; (**c**) the C_f_/PyC interphase with ${\bar{L}}_{a}$ equal to 8 nm-∞.

Using the MD simulation model of the C_f_/PyC interphase established in Section 2.2.1, the normal (z direction) tensile properties with different ${\bar{L}}_{a}$ were calculated at room temperature (300 K). In each calculation, graphite sheets within 1.5 nanometers from the top and bottom of the simulation box were defined as the top and bottom boundaries, respectively (including those sheets that were partially within the range). The bottom boundary atoms were fixed by setting the velocity equal to 0. Meanwhile, the velocity of the top boundary atoms was set to *V* in the Z direction and equal to 0 in the other two directions. *V* was calculated as follows:

(2) $$V=0.001*L_{Z-\mathrm{load}}$$

where *L*_*Z*-load_ is the Z directional length of the simulation box except for boundary regions. With a time step of 1 fs, the tensile strain increased 0.001 every 1000 steps. The volume-average virial stress of all atoms, except those in boundary regions, was calculated every 1000 steps to form the stress–strain relationship. The typical normal tensile stress–strain curves are shown in Figure 5b,c, respectively.

**Figure 6:**
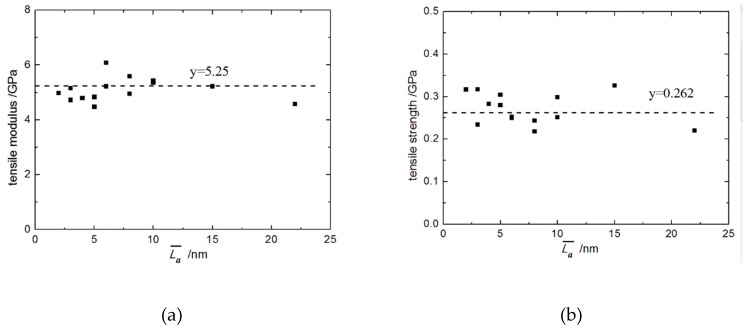
The normal tensile mechanical properties of the C_f_/PyC interphase with different sizes of ${\bar{L}}_{a}$: (**a**) Modulus; (**b**) Strength.

In addition, using a hexagonal graphite MD model with 20 sheets and 64,000 atoms, the out-of-plane tensile stress–strain response of perfect hexagonal graphite was calculated by the same loading process, and is drawn in Figure 5a. The calculated normal tensile modulus and strength of perfect graphite are 23.33 GPa and 1.16 GPa, respectively. Meanwhile, the interfacial normal tensile modulus and strength of the C_f_/PyC interphase with different ${\bar{L}}_{a}$ are plotted as shown in Figure 6a,b. The progressive line (the dotted line) in Figure 6 is the calculation result when ${\bar{L}}_{a}=\infty$.

As shown in Figure 5, most of the stress–strain curves with different ${\bar{L}}_{a}$ have bilinear characteristics, but a few have a longer platform after reaching the ultimate strength. Compared with the stress–strain response of perfect hexagonal graphite, the fracture strain does not change significantly, while there are sharp declines in both the tensile modulus and strength. On the other hand, as can be seen from Figure 6, as the size of ${\bar{L}}_{a}$ changes ($2\;\mathrm{nm}\leq {\bar{L}}_{a}\leq 22\;\mathrm{nm}$), the fluctuations of both the tensile modulus and strength are all centered on the numerical results calculated when ${\bar{L}}_{a}=\infty$. Therefore, the value of ${\bar{L}}_{a}$ has little effect on the C_f_/PyC interfacial normal tensile mechanical properties.

#### 3.1.2. Failure Mechanism of the C_f_/PyC Interphase under a Normal Tensile Load

**Figure 7:**
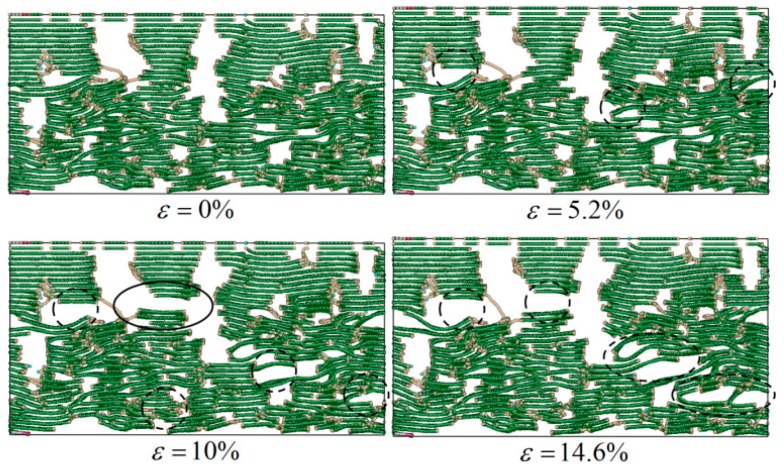
Typical failure modes of the C_f_/PyC interphase under a normal tensile load when ${\bar{L}}_{a}=3\;\mathrm{nm}$.

**Figure 8:**
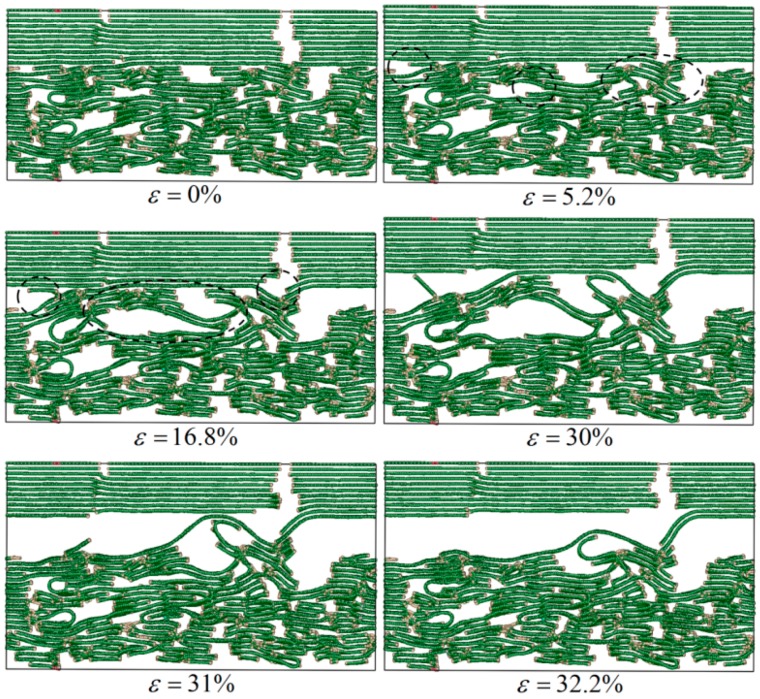
Typical failure modes of the C_f_/PyC interphase under a normal tensile load when ${\bar{L}}_{a}=10\;\mathrm{nm}$.

The above laws are mainly related to the deformation and failure mechanism of the C_f_/PyC interphase under a normal tensile load. For a small (e.g., 3 nm) and a large (e.g., 10 nm) ${\bar{L}}_{a}$ value, the failure modes of the C_f_/PyC interphase under a normal tensile load are shown in Figure 7 and Figure 8, respectively.

As shown in Figure 7, when ${\bar{L}}_{a}$ is small, in the PyC region there exists a large number of short graphite sheets that do not arrange along the longitudinal (x direction) of the fiber. There are some long sheets that even cross several layers in the normal direction (z direction). These sheets form voids with the surrounding graphite wafers, and the bonding between crystals is relatively weaker due to the longer distance. Therefore, under normal tensile loading, these sheets will rotate under the action of torque, the original micro-voids will expand rapidly (as marked with the dotted line in Figure 7 when ε=5.2%), and the interaction will be lost first in these regions (as marked with the solid line in Figure 7 when ε=10%). Finally, obvious large voids occur all along the x direction (as marked with the dotted line in Figure 7 when ε=14.6%); thus, the bearing capacity is lost.

Figure 8 shows the failure process of the C_f_/PyC interphase under a normal tensile load when the value of ${\bar{L}}_{a}$ is large. The failure process is similar to that when ${\bar{L}}_{a}$ is smaller. The structural damage still begins with the deflection of some graphite sheets, resulting in the expansion of the original micro-voids (as marked with the dotted line in Figure 8 when ε=5.2%). Finally, the entire structure fails (as marked with the dotted line in Figure 8 when ε=16.8%).

**Figure 9:**
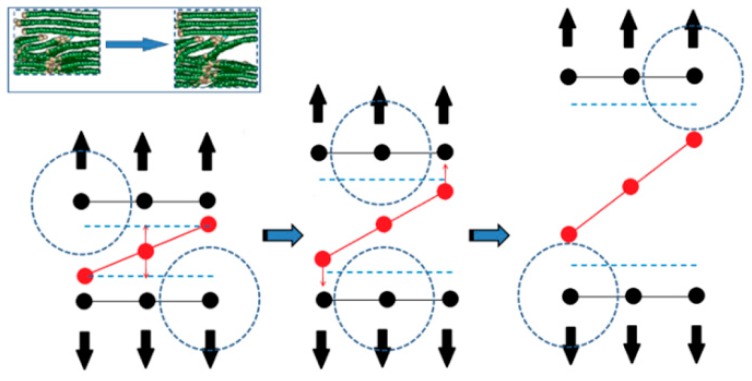
The typical failure mode of the C_f_/PyC interphase under a normal tensile load.

To sum up, the rotation of deflected graphite sheets is the main failure mechanism of the C_f_/PyC interphase under a normal tensile load, as shown in Figure 9. It is also the main reason for the sharp declines in both the tensile modulus and strength compared with the perfect graphite structure. Since the failure mechanism and process do not change significantly with different ${\bar{L}}_{a}$, the ${\bar{L}}_{a}$ value has no obvious influence on the normal tensile mechanical properties of the C_f_/PyC interphase.

### 3.2. MD Simulation Results of the C_f_/PyC Interphase under Tangential Shear

#### 3.2.1. Interfacial Tangential Shear Property with Different ${\bar{L}}_{a}$

**Figure 10:**
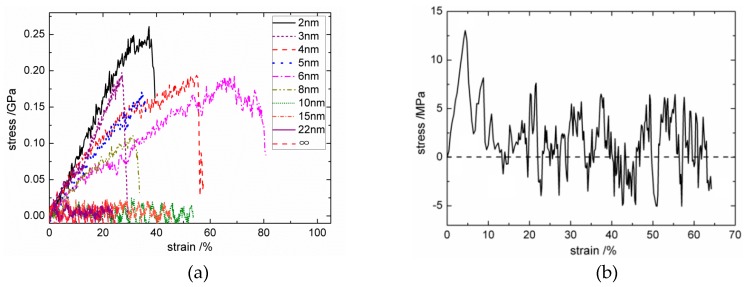
Typical tangential shear stress–strain curves: (**a**) the C_f_/PyC interphase with different sizes of ${\bar{L}}_{a}$; (**b**) perfect hexagonal graphite.

**Figure 11:**
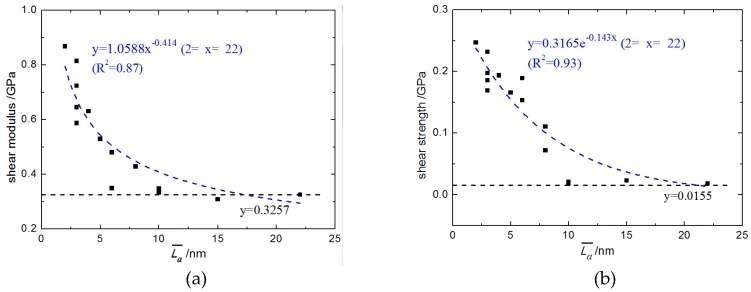
The tangential shear mechanical properties of the C_f_/PyC interphase with different sizes of ${\bar{L}}_{a}$: (**a**) Modulus; (**b**) Strength.

The MD simulation process of both the C_f_/PyC interphase and perfect hexagonal graphite under tangential shear was similar to that under a normal tensile load, except that the velocity *V* setting for top boundary atoms was in the X direction. Typical tangential shear stress–strain curves are shown in Figure 10. The tangential shear modulus and strength of the interphase with different values of ${\bar{L}}_{a}$ are drawn in Figure 11a,b, respectively. The calculated shear modulus and strength of perfect hexagonal graphite were 0.431 GPa and 0.013 GPa, respectively. The horizontal asymptotic line (the dotted line) in Figure 11 is the calculation result when ${\bar{L}}_{a}=\infty$.

As shown in Figure 10, when ${\bar{L}}_{a}$ is relatively small ($2\;\mathrm{nm}\leq {\bar{L}}_{a}\leq 8\;\mathrm{nm}$), the shear stress–strain curves show obvious brittle fracture characteristics. However, when ${\bar{L}}_{a}$ becomes larger (${\bar{L}}_{a}\geq 10\;\mathrm{nm}$), the shear strength of the C_f_/PyC interphase decreases obviously. To be specific, when ${\bar{L}}_{a}\geq 10\;\mathrm{nm}$, the shear stress–strain curves reach the ultimate strength under small strain, and then enter an approximately constant amplitude oscillation stage, which is similar to the response of perfect hexagonal graphite. As shown in Figure 11, the interfacial shear modulus and shear strength have obvious regularity with the change in ${\bar{L}}_{a}$. After fitting the calculated data points, it was found that the variation law of interfacial shear modulus *G*_S_ with the average length of fiber surface sheets ${\bar{L}}_{a}$ approximately satisfies the power function:

(3) $$G_{S}=1.0588{\bar{L}}_{a}^{-0.414}\;(2\leq {\bar{L}}_{a}\leq 22).$$

Meanwhile, the variation law of interfacial shear strength *S*_S_ with the average length of fiber surface sheets ${\bar{L}}_{a}$ approximately satisfies the exponential relation:

(4) $$S_{S}=0.3165e^{-0.143{\bar{L}}_{a}}\;(2\leq {\bar{L}}_{a}\leq 22).$$

#### 3.2.2. Failure Mechanism of the C_f_/PyC Interphase under Tangential Shear

**Figure 12:**
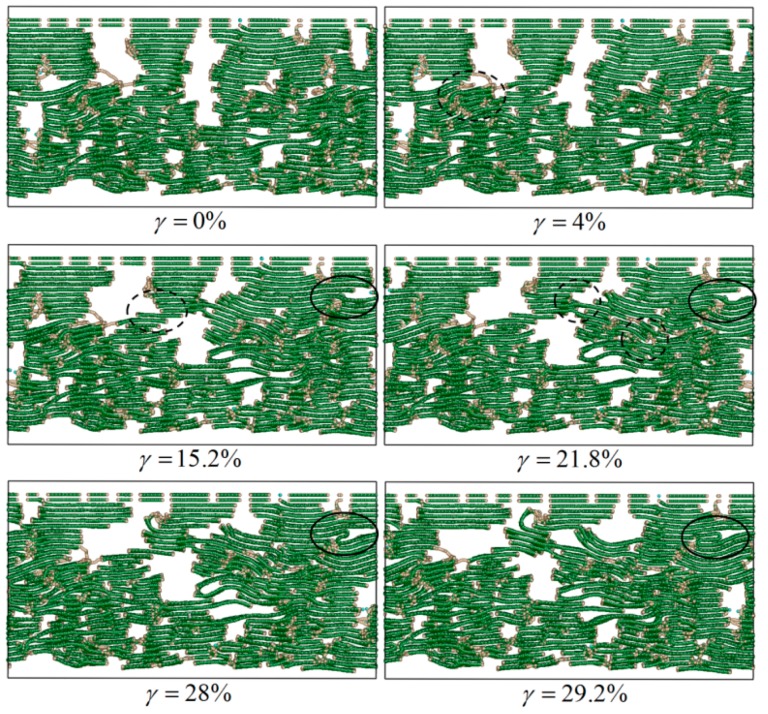
Typical failure modes of the C_f_/PyC interphase under a tangential shear load when ${\bar{L}}_{a}=3\;\mathrm{nm}$.

**Figure 13:**
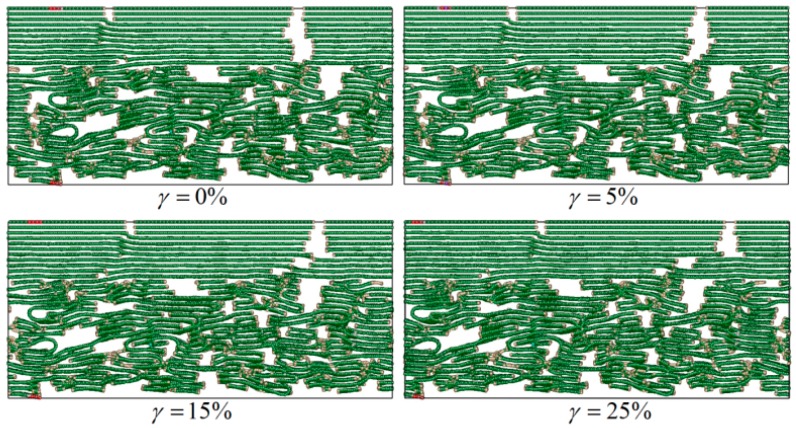
Typical failure modes of the C_f_/PyC interphase under a tangential shear load when ${\bar{L}}_{a}=10\;\mathrm{nm}$.

In order to investigate the influence of ${\bar{L}}_{a}$ on the shear mechanical properties of the C_f_/PyC interphase, failure behaviors are compared between ${\bar{L}}_{a}$ being small (taking 3 nm as an example) and large (taking 10 nm as an example), as shown in Figure 12 and Figure 13, respectively.

**Figure 14:**
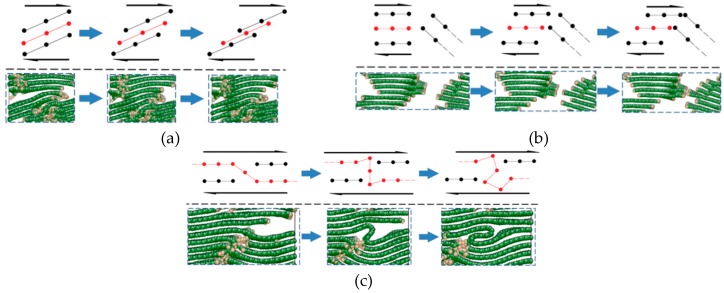
The main mechanism of the C_f_/PyC interphase to improve the shear mechanical properties when ${\bar{L}}_{a}$ is smaller: (**a**) non-in-plane shear behavior; (**b**) the barrier between crystals; (**c**) long sheet folding.

As shown in Figure 12, when ${\bar{L}}_{a}$ is small, the distribution of graphite sheets in both the fiber surface and the PyC region nearby is disordered. In some regions, since the sheets are not arranged along the longitudinal direction of the fiber (x direction) (as marked with the dotted line in Figure 12 when γ=4%), with the increase in the tangential shear load, the distance between graphite sheets decreases, leading to a stronger bonding force between adjacent graphite sheets. Therefore, it is more difficult for the layers to slide, and the shear resistance of the whole structure increases (as shown in Figure 14a). Meanwhile, when the orientation of the adjacent graphite crystals is inconsistent (as marked with a dotted line in Figure 12 when γ=21.8%), it will also hinder the relative slip; thus, the shear strength of the structure further increases (as shown in Figure 14b). In addition, due to the existence of long sheets that cross several layers in the normal direction (z direction), bending, folding, and deformation occurs in these long sheets under the tangential shear load. This absorbs more energy, hinders the relative movement of the nearby crystals, and makes the shear property of the structure further increase (as marked with a solid line in Figure 12 and shown in Figure 14c).

On the other hand, as shown in Figure 13, when ${\bar{L}}_{a}$ is large, the orientation of the graphite sheets in the fiber surface is higher. Therefore, under the tangential shear load, the main deformation and failure mode of the C_f_/PyC interphase is the relative slip between the large graphite sheets in the fiber surface region, while the PyC region has a small amount of deformation. In this way, the tangential shear properties of the entire C_f_/PyC interphase mainly depend on the sliding behavior between the large graphite sheets in the fiber surface, which is similar to the deformation mode of perfect graphite crystal. Therefore, the interfacial shear properties with a larger ${\bar{L}}_{a}$ are lower.

To sum up, when the value of ${\bar{L}}_{a}$ is small, there are a large number of slanting graphite sheets both in the fiber surface and the PyC region nearby, together with long crystals that cross several layers in the normal direction (z direction). Under the tangential shear load, the shear modulus and shear strength of the C_f_/PyC interphase are greatly improved due to the effects of non-in-plane shear behavior, the barrier between crystals, and the long sheet folding. As the value of ${\bar{L}}_{a}$ increases, the interfacial shear modulus and strength decrease. When ${\bar{L}}_{a}$ increases to a certain extent, the main shear failure mechanism of the C_f_/PyC interphase changes to the relative slip between large crystal sheets in the fiber surface region. At this time, the interfacial shear modulus and strength are low, and no longer change significantly with the increase in ${\bar{L}}_{a}$.

### 3.3. MD Simulation Results of the IPyC Matrix under Tangential Shear

**Figure 15:**
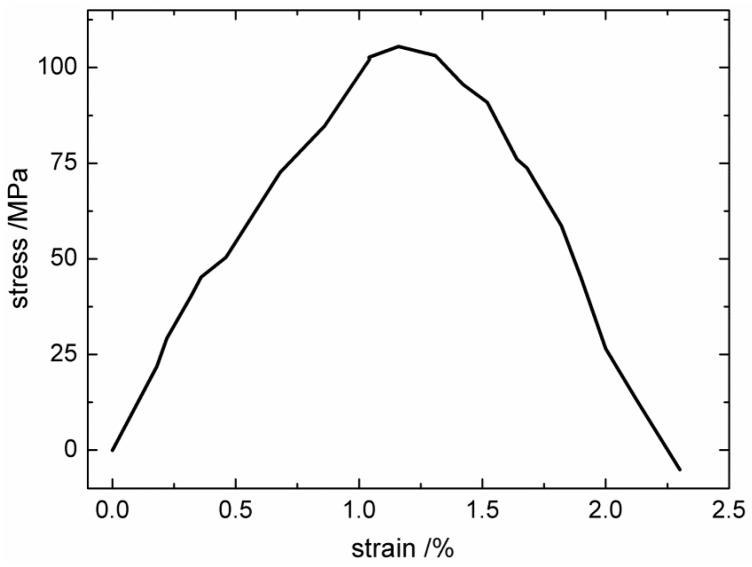
The tangential shear stress–strain curve of the isotropic pyrolysis carbon (IPyC) matrix.

Using the same simulation process mentioned in Section 3.2.1, the tangential shear stress–strain curve of the IPyC matrix was obtained, and is shown in Figure 15.

As shown in Figure 15, the calculated shear stress–strain curve of the IPyC matrix also has the bilinear characteristic. Since hybrid forms of both sp^2^ and sp^3^ exist in the IPyC matrix, the shear properties of IPyC are much higher than those of perfect hexagonal graphite (which will slide between graphite sheets under in-plane shear). The calculated shear modulus *G*_IPyC_ = 9.8 GPa, and the shear strength *S*_IPyC_ = 105 MPa.

### 3.4. Verification of MD Simulation Results

In order to verify the validity of the MD simulation results, based on the experimental conditions reported by Long et al. [21], a finite element analysis (FEA) was carried out to simulate the C/C composite fiber ejection test at the micron scale. The experimental conditions in [21] are summarized as follows: the average diameter of T300 carbon fiber was 7 μm, and the density of the IPyC matrix was 1.995 g/cm^3^. C/C composites were processed into experimental pieces with a thickness of 84 μm. The test was carried out on the nano indentation tester using a diamond pressure head. The head radius was 0.3 times that of the fiber. The bottom of the test specimen was held by an aluminum base.

#### 3.4.1. Establishment of the FEA Model

**Figure 16:**
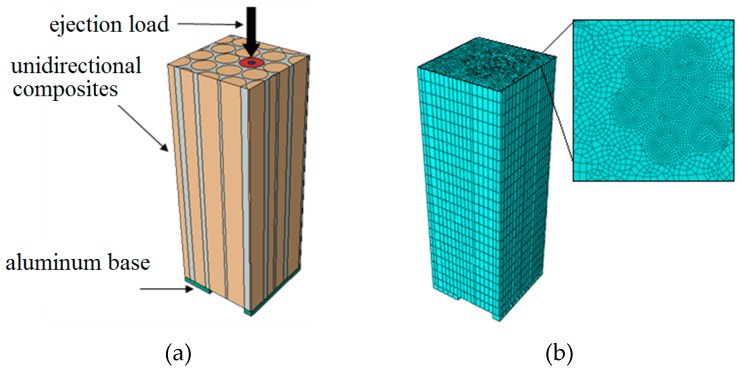
The simulation model of the fiber ejection test on the carbon/carbon (C/C) composite: (**a**) geometric model; (**b**) finite element model.

The (Computer-Aided Design) CAD model established in this paper is shown in Figure 16a. The C/C unidirectional composite cell was built by using the collision algorithm [22] to ensure both the characteristics of the random fiber distribution and the periodic repeatability requirement on the boundary. Periodic boundary conditions were applied around the cell. Contact was set at the bottom of the composite to simulate the interaction between the specimen and the aluminum base. A downward ejection displacement load was applied to the upper end of the ejected fiber, and the radius of the applied area was 0.3 times that of the fiber radius. Hexahedral mesh was used, and local refinement of the mesh was carried out in the pushed-out fiber and its adjacent area, as shown in Figure 16b.

Since both the tensile and shear stress–strain responses of the C_f_/PyC interphase satisfied the bilinear constitutive relation, the contact element containing the cohesive force model and the Traction–Separation bilinear constitutive were used to characterize the mechanical properties of the interphase [23]. It can be obtained from [18,24] that the average length of graphite sheets in the surface of T300 carbon fiber is 2~4 nm. The median value ${\bar{L}}_{a}$ = 3 nm was taken in formulas (3) and (4) to calculate the shear modulus (*G*_S_) and the shear strength (*S*_S_) of the C_f_/PyC interphase, respectively. The normal tensile modulus (*K*_T_) and strength (*S*_T_) were set according to the mean value of the calculated results in Section 3.1.1, and are listed in Table 1. Based on the calculated results in Section 3.3, the properties of the IPyC matrix used in the FEA are also listed in Table 1. The properties of T300 carbon fiber were obtained from [25], and are listed in Table 2. The maximum stress criterion was used as the failure criterion for both the interphase and the matrix, while fiber failure and aluminum base failure were not considered.

#### 3.4.2. FEA Results of the Fiber Ejection Test Simulation

**Figure 17:**
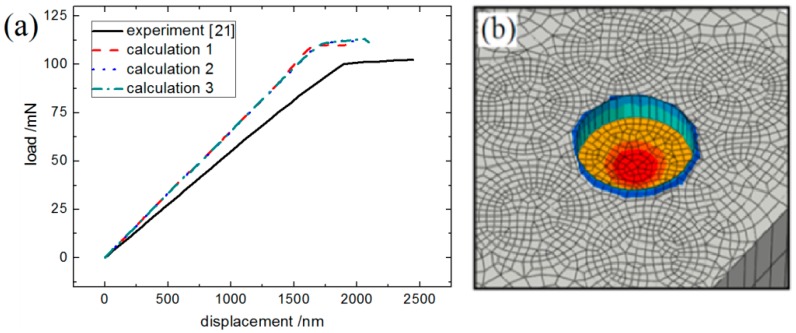
A comparison between the simulation and experimental results of the C/C composite fiber ejection test: (**a**) the load–displacement curves; (**b**) the deformation of the ejected fiber in the simulation.

Three carbon fibers near the center of the model were selected for analysis. The simulated load–displacement curves, together with the experimental one reported in [21], are shown in Figure 17a. The simulated deformation of the fiber after ejection is shown in Figure 17b.

As shown in Figure 17a, the calculated load–displacement curves are in good agreement with the experimental test results. From Figure 17b, the fiber after ejection presents obvious slip deformation, while the fiber itself remains intact. The deformation behavior of the ejected fiber and the nearby matrix region obtained by simulation is consistent with the observed morphology in literature [27]. The maximum load value *P* and the interfacial nominal shear strength (IFSS) obtained by both experiment and simulation are listed in Table 3, in which the IFSS is calculated as follows:

(5) $$IFSS=\frac{P}{\pi dt}$$

where *P* is the maximum load, *d* is the fiber diameter, and *t* is the thickness of the ejected specimen. As can be seen from Table 3, the simulation results of this paper are in good agreement with the experimental test results, which proves the validity of the research method and results in this paper.

## 4. Conclusions

In this paper, the MD simulation method was used to calculate the CVI deposition process of a PyC matrix onto a carbon fiber surface under certain assumptions. The obtained MD simulation model well-represented the true microstructure of the C_f_/PyC interphase. On this basis, the mechanical properties and failure mechanism of the C_f_/PyC interphase under tangential shear and a normal tensile load were studied with different sizes of ${\bar{L}}_{a}$, while the influence of ${\bar{L}}_{a}$ on the interfacial mechanical properties and failure mechanism was obtained. The shear properties of the IPyC matrix were also presented by MD simulation. Based on the MD simulation results, the fiber ejection test of C/C composites at a micron scale was simulated by the FEA method, and the correctness of the MD simulation results was verified. Through the research in this paper, the following conclusions were obtained:

(1) Under a normal tensile load, due to the existence of deflected graphite sheets, the structural damage to the C_f_/PyC interphase is mainly caused by the rotation of these deflected graphite sheets, which results in the expansion of the original micro-voids and leads to the interruption of force transmission paths. ${\bar{L}}_{a}$ has no obvious influence on the C_f_/PyC interfacial normal tensile mechanical properties (initial modulus and ultimate strength).

(2) Under a tangential shear load, when the size of ${\bar{L}}_{a}$ is small, due to the effects of non-in-plane shear, a barrier between crystals, and the long sheet folding, the shear performance of the C_f_/PyC interphase is relatively high. As ${\bar{L}}_{a}$ increases, the shear failure mode gradually turns into the relative slip between the large sheets in the fiber surface, and the shear performance of the interphase decreases and converges to the value when ${\bar{L}}_{a}=\infty$. The variation law of interfacial shear modulus *G*_S_ with ${\bar{L}}_{a}$ approximately satisfies the power function, while the variation law of interfacial shear strength *S*_S_ with ${\bar{L}}_{a}$ approximately satisfies the exponential relation.

## Figures and Tables

**Table 1:** The mechanical properties of the C_f_/PyC interphase (${\bar{L}}_{a}$ = 3 nm) and the IPyC matrix.

C_f_/PyC Interphase	*K*_T_/GPa	*G*_S_/GPa		*S*_T_/GPa		*S*_S_/GPa
	4.94	0.67		0.55		0.2
IPyC matrix	*G*/GPa		*ν*		*S*/MPa	
	9.8		0.23 [26]		105	

**Table 2:** The elastic properties of the T300 carbon fiber and aluminum base [25].

T300 Fiber	*E*_11_/GPa	*E*_22_ = *E*_33_/GPa	*G*_12_ = *G*_13_/GPa	*G*_23_/GPa	$\nu_{12}=\nu_{13}$	$\nu_{23}$
	220	22	4.8	7.7	0.12	0.42
aluminum base	*G*/GPa				*ν*	
	70				0.33	

**Table 3:** A comparison of the experimental and simulated results of fiber ejection on the C/C composite.

	t/μm	*P*/mN	IFSS/MPa
Experiment [21]	84	97 ± 7	53 ± 4
simulation	84	111.7	60.5
Error (%)	-	7%–23%	7%–23%

IFSS, interfacial nominal shear strength.
